# Occupational heat stress in hospital settings: a combined assessment of staff perceptions and indoor climate to support heat action planning

**DOI:** 10.1186/s12889-026-28452-4

**Published:** 2026-07-08

**Authors:** Sophie Theresia Scheidl, Irena Kaspar-Ott, Elke Hertig

**Affiliations:** Regional Climate Change and Health, Faculty of Medicine, University of Augsburg, Universitätsstraße 2, Augsburg, 86159 Germany

**Keywords:** Occupational health, Hospital settings, Indoor heat exposure, Healthcare workers, Climate change adaptation, Heat action planning

## Abstract

**Background and aims:**

Occupational heat stress in healthcare settings is an emerging public health concern, yet evidence on indoor heat exposure and perception in hospitals remains limited. This study assessed perceived occupational heat stress among hospital employees by integrating staff-reported heat perceptions with indoor temperature measurements to identify associations between perceived heat stress, workplace characteristics, preventive behaviors, heat-related knowledge, and indoor thermal conditions.

**Methods:**

A cross-sectional study was conducted at a large tertiary care hospital in southern Germany. An anonymous online survey collected data on heat perception, heat-related symptoms, work impairment, preventive behaviors, knowledge, training and heat warning practices among hospital employees. Multiple survey items resulted in a perceived occupational heat stress index (POHSI). Indoor air temperature was measured continuously during the summer periods 2024 and 2025 using calibrated sensor loggers placed in staff rooms across the hospital. Associations between POHSI, workplace factors, preventive measures, and environmental conditions were analyzed descriptively and inferentially.

**Results:**

A total of 681 employees participated. 38.7% of the participants scored high or very high POHSI. Frequently reported symptoms included fatigue, concentration difficulties, and dizziness, accompanied by reduced work performance. Higher POHSIs were observed among physically active staff and clustered spatially in upper floors with elevated indoor temperatures. Lower levels of heat-related knowledge and training were associated with higher POHSIs (ANOVA: F = 15.33, *p* < 0.001; Kruskal–Wallis: χ² = 54.05, *p* < 0.001) with moderate effect sizes (η² ≈ 0.09). While some individual preventive measures were associated with lower POHSI, participants predominantly requested structural and organizational interventions. Subjective heat perceptions corresponded closely with measured indoor temperature patterns.

**Conclusion:**

Occupational heat stress represents a relevant indoor environmental and occupational health challenge in hospital settings. The combination of subjective and objective indicators enabled the identification of heat-vulnerable work environments and associated workplace, knowledge-related, and environmental factors. The findings further indicate that individual coping strategies alone may be insufficient under sustained indoor heat exposure and support the development of hospital-specific heat prevention and adaptation strategies.

**Supplementary Information:**

The online version contains supplementary material available at 10.1186/s12889-026-28452-4.

## Introduction

Heat extremes are increasing in frequency, intensity, and duration due to climate change and represent a growing public health challenge [1–3]. Heat exposure is associated with adverse health effects reducing physical and mental performance [2]. In occupational settings, heat stress has been linked to increased error rates, productivity losses and increased work-related accidents, particularly in physically or cognitively demanding professions [4, 5]. In Germany, annual productivity losses attributable to rising heat exposure are projected to reach approximately 6.9 million working hours by the end of the century [6].

Healthcare systems constitute critical public infrastructure that must remain functional during heat events while responding to increased demand for care related to heat-associated illnesses [7]. Hospital staff therefore face a dual burden during heat events: elevated occupational heat exposure and increased workload. Physically demanding tasks, time pressure and shift work increase staff vulnerability [8, 9] Addressing occupational heat stress in hospitals is thus a public health priority, with implications for healthcare professionals, patients and healthcare system resilience.

While occupational heat stress has been widely studied in outdoor work and industrial settings, evidence in hospital settings remains limited, particularly in German-speaking countries [10–12] Available research suggests that thermal conditions in hospital buildings are shaped by the interaction of structural characteristics such as building age, insulation, floor level and orientation, alongside internal heat loads, ventilation options and work activities [11, 12].

Many hospitals, especially older facilities, rely primarily on mechanical ventilation and lack comprehensive air conditioning outside critical areas such as operating theatres or intensive care units [13]. During heat events, indoor temperatures may therefore rise substantially, leading to prolonged heat exposure for staff. Despite the relevance of indoor thermal conditions in hospital workplaces, systematic data on indoor thermal environments in hospitals and their effects on staff health and work performance remain scarce, limiting the evidence base for heat prevention strategies [14].

Heat exposure is not determined solely by environmental parameters but is strongly influenced by individual perception. Subjective indoor heat perception combines ambient temperature, humidity, workload, clothing, individual susceptibility and adaptive capacity [15]. Perceived heat stress is closely associated with symptom burden, cognitive performance and productivity loss [5, 16, 17].

In indoor occupational settings, discrepancies may exist between objectively measured thermal conditions and perceived heat burden, particularly under high work demands [8]. Incorporating subjective heat perception alongside objective measurements may improve the identification of heat-vulnerable workplaces and support worker-centered prevention strategies.

The study aimed to assess perceived occupational heat stress and related symptoms among hospital employees, examine associations between subjective heat burden, workplace factors, preventive behaviors, and indoor thermal conditions, and identify spatial patterns of occupational heat burden to inform hospital heat prevention planning.

## Methods

### Setting and study design

Questionnaire data from hospital employees were combined with objective environmental temperature and humidity measurements across the University Hospital Augsburg (Universitätsklinikum Augsburg, UKA) within a cross-sectional observational study. This design allowed us to assess real-world occupational heat exposure.

This study was conducted at the UKA, a tertiary care hospital in southern Germany with approximately 7,500 employees and 1,700 beds across more than 24 clinical departments. Temperature and humidity measurements primary focused on the main hospital building (14 floors) opened in 1982. The building uses mechanical ventilation without central air conditioning, except in specific areas (e.g. operation theaters). Upper floors (10–12) and south- or west-facing rooms are known heat-vulnerable areas.

### Participants and recruitment

All employees were invited to participate in an anonymous online survey hosted on SoSci Survey (Version 3.6.06) [18]. The German questionnaire was accessible from 2nd May to 30th June 2025 and comprised 23 items, taking approximately 10 min to complete. Recruitment occurred on institution-wide communication channels. Participation was voluntary and uncompensated. Due to the exploratory nature of the study and the open invitation strategy, no formal sample size calculation was performed. The Ethics Committee of LMU Munich determined that no formal consultation was required (Project No. 24-1017-KB).

### Survey development, content and validation

The questionnaire aimed to assess subjective heat perception, related symptoms, preventive behaviors and knowledge among hospital employees. Survey items were informed by a review of occupational and perceived heat stress literature [5, 12, 19–21], adapted and supplemented to the setting. Draft items were sequentially reviewed for clarity by colleagues, for relevance by environmental health experts, and for contextual applicability by hospital staff, including management, occupational health and human resources specialists. This structured expert review provided content validation and guided minor revisions before administration.

The final questionnaire covered 23 items across six domains: (1) demographic and work-related characteristics; (2) heat perception and thermal discomfort; (3) heat-related symptoms and work impairment; (4) currently implemented and desired preventive measures; (5) knowledge, training and preparedness for heat events; and (6) heat warning systems and information sources. Items used 5-point Likert scales, multiple-choice, or yes/no response formats, with selected open-ended questions to capture additional responses (translated survey see Supplements S1).

Domains were selected based on evidence that subjective heat perception predicts heat strain and productivity loss [8, 12, 21], that heat exposure can cause specific symptoms [2, 8, 22], and that knowledge, training and warning influence adoption of preventive behaviors [12, 19, 23, 24]. Internal consistency was evaluated using Cronbach’s alpha, inter-item correlations and principal component analysis.

### Development and internal consistency of the perceived occupational heat stress index (POHSI)

A composite POHSI was calculated by summing responses from five items assessing heat: perception (BL01), frequency of exposure (BL03), impact on work productivity (BL04), number of heat-related symptoms (BL05) and inability to work (BL06). All items were equally weighted. Likert-scale responses ranged from 0 (“not at all”) to 4 (“very strong”). Symptom counts were rescaled to a 0–4 range to ensure comparability. The resulting POHSI ranged from 0, indicating no perceived heat stress or work impairment, to 20, indicating maximal perceived heat stress and severe work-related effects. Scores were classified as very low (0–4), low (5–8), medium (9–12), high (13–16), very high (17–20).

The POHSI was calculated as follows:

$$\:{\mathrm{POHSI}}_{\boldsymbol{i}}={\mathrm{BL01}}_{\boldsymbol{i}}+{\mathrm{BL03}}_{\boldsymbol{i}}+{\mathrm{BL04}}_{\boldsymbol{i}}+(\frac{{\mathrm{BL05}}_{\boldsymbol{i}}}{9}\times\:5)+{\mathrm{BL06}}_{\boldsymbol{i}}$$

Internal consistency was evaluated using Cronbach’s alpha and item-total correlations. Distribution was assessed using descriptive statistics, including mean, standard deviation, skewness, kurtosis, and normality tests (Shapiro–Wilk).

### Indoor temperature measurements

Indoor air temperature (°C) was measured to characterize thermal workplace conditions and to support interpretation of subjective heat perceptions. Furthermore, relative humidity (%) was measured to capture the combined discomfort due to high temperatures and humidity (sultriness). Measurements were conducted using low-cost sensor loggers installed in staff rooms across the UKA, which have been shown to provide reliable indoor climate data [25] 15 sensors were deployed in 2024, two sensors added in 2025, on different floor levels of the main hospital building and the lower floor of the Mother–Child Center (MCC), reflecting typical staff work environments and known heat-vulnerable areas (see S2 in Supplements). Sensor locations were selected to capture spatial variation in indoor thermal conditions across different floor levels, building areas and orientations, and perceived heat-prone locations within the hospital. Sensors were positioned at approximately 1.1 m height and protected from direct sunlight and heat sources to improve comparability of indoor measurements.

Sensors recorded air temperature and relative humidity continuously at 4-second intervals between 15th May 2024 and end of October 2025. For analysis, data were restricted to the summer periods 15th May to 15th October for both years. All sensors were calibrated in-house against a precision reference system (ALMEMO^®^, Ahlborn GmbH) using a one-week co-location procedure. Measurements were recorded at 4-second intervals. The mean temperature difference relative to the reference device functioned as a constant additive offset correction.

### Statistical analysis

Descriptive statistics summarized demographics, workplace variables and survey responses using frequencies and percentages (categorical variables) and means with standard deviations for continuous variables. Associations between categorical variables and participant characteristics were examined using chi-square tests with Monte Carlo simulation (10,000 replications). Effect sizes were calculated using Cramér’s V. To control for multiple testing in symptom-related analyses, p-values were adjusted using the Benjamini–Hochberg false discovery rate procedure.

POHSI distributions were inspected statistically (Shapiro–Wilk test). Group differences were assessed using independent-samples t-tests and one-way analysis of variance (ANOVA) or Kruskal–Wallis tests. Post hoc pairwise comparisons were adjusted for multiple testing. Associations with ordinal or continuous variables were examined using Spearman’s rank correlation coefficients. All tests were two-sided, and a p-value < 0.05 was considered statistically significant.

Temperature data were analyzed descriptively, including calculation of means, minima and maxima. To facilitate comparison with survey-reported heat hotspots and identify spatial patterns within the hospital environment, sensor locations were categorized into temperature quartiles based on box-wise means. The lower and upper quartiles therefore represent comparatively cooler and warmer areas within the hospital.

Statistical analyses were performed using R (version 4.5.1).

## Results

### Study participation

A total of 681 employees participated, of whom 607 completed the survey, corresponding to a response rate of approximately 9% of 7,500 total employees. Most respondents were aged 25–45 years (55%), with 9.6% under 25. Most participants were female (76%). Participating employees reported working in clinical roles (doctors, nurses and similar) in 65% of cases, non-clinical roles (administration, management, IT etc.) in 34%. Participant demographics are summarized in Table 1, full details are provided in Supplements S3.

**Table 1:** Demographic characteristics of the study participants

Characteristic	*N* = 681^1^
Age	
16–25 Years	65 (9.6%)
26–35 Years	224 (33%)
36–45 Years	149 (22%)
46–55 Years	131 (19%)
56–65 Years	100 (15%)
Over 65 Years	3 (0.4%)
Gender	
female	517 (76%)
male	148 (22%)
divers	2 (0.3%)
Occupational Category	
Clinical	440 (65%)
Non-clinical	234 (34%)

^1^n (%) per category

### Heat perception and POHSI

**Fig. 1:**
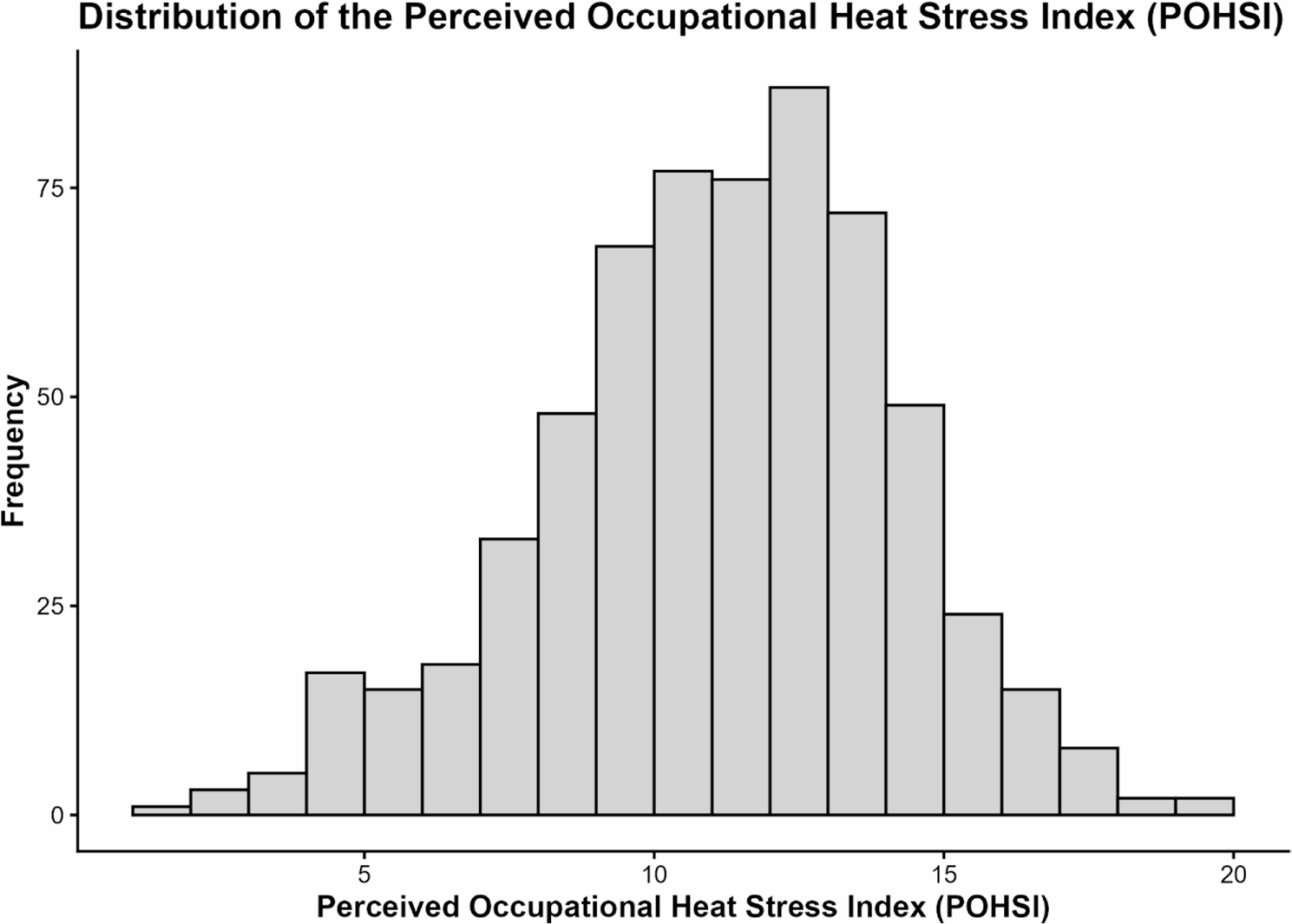
Distribution of the Perceived Occupational Heat Stress Index (POHSI) among study participants

Survey responses indicate that 50% of the participants perceive temperatures during summer at their workplace as too hot, 27% as hot and 10% as pleasant. 1% reported temperature as cold at their workplace. Calculation of the POHSI showed that most respondents scored an average POHSI (40.9%), 34.3% scored a high POHSI, and 4.4% scored a very high POHSI. 13.9% scored a low or very low POHSI, see Fig. 1. The POHSI showed a mean value of 11.13 (SD = 3.04) with a range from 1.44 to 19.11.

The distribution showed minimal deviation from normality (skew = − 0.33, kurtosis = 0.13), justifying the use of parametric analyses assuming normal distribution. The scale showed good internal consistency (Cronbach’s α = 0.79, 95% CI: 0.76–0.81). The average inter-item correlation was *r* = 0.43.

### Heat-related symptoms and work impairment

Participants commonly reported neurological, thermoregulatory, cardiovascular, and exhaustion-related symptoms (Supplementary S4). Significant associations were observed between symptoms and demographic and work-related factors. Dizziness and headaches were reported more frequently by younger participants (< 35 years) (p_adj = 0.001; Cramér’s V = 0.225 and 0.194, respectively). Clinically active participants showed a higher prevalence of excessive sweating (p_adj = 0.0182; Cramér’s V = 0.127). Several symptoms were associated with physically active occupations, including excessive sweating, dizziness, exhaustion, palpitations, and headaches (all p_adj < 0.005; V = 0.162–0.230). In contrast, sedentary participants reported symptoms less frequently (p_adj = 0.003; V = 0.183). Effect sizes were small to medium.

Heat exposure substantially affected perceived work performance, particularly among clinical staff. A very strong impact was reported by 17.8% of respondents, corresponding to 20.4% of clinical and 13.1% of non-clinical participants. A high impact was reported by 45.0% overall (46.2% clinical, 43.0% non-clinical), while 29.0% reported a moderate impact. The perceived impact of heat on work performance differed significantly by gender, age and physical activity level (all *p* < 0.001, Cramér’s V: 0.502–0.519), as well as occupational role (*p* < 0.001; Cramér’s V = 0.429).

### Preventive measures

**Fig. 2:**
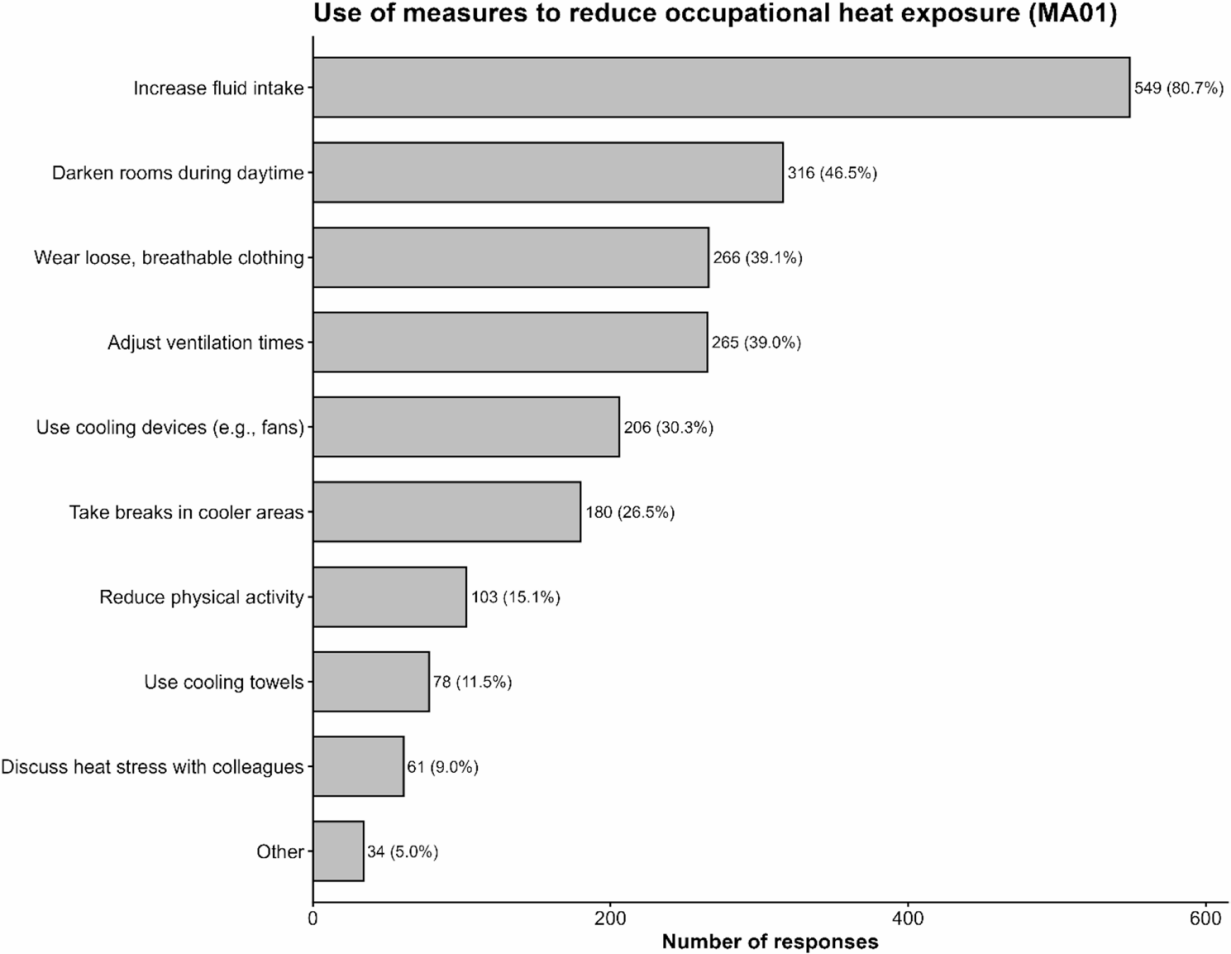
Measures currently being implemented by participants to reduce occupational heat exposure. Bars represent the number of responses for each measure, with values shown as n (%)

Current preventive measures are summarized in Fig. 2. Preventive behaviors were broadly similar across gender and occupational groups, although some differences in the type of measures implemented were observed.

Male participants more frequently reported taking breaks in cooler areas (*p* = 0.002; Cramér’s V ≈ 0.12) and reducing physical activity (*p* < 0.001; Cramér’s V ≈ 0.13).

Occupational category was associated with wearing loose clothing (χ² = 110.0, *p* < 0.001; Cramér’s V = 0.424), reducing physical activity (χ² = 15.3, *p* < 0.001; Cramér’s V = 0.155), darkening rooms (χ² = 5.29, *p* < 0.02; Cramér’s V = 0.09) and adjusting ventilation times (χ² = 10.2, *p* = 0.001; Cramér’s V = 0.127).

**Fig. 3:**
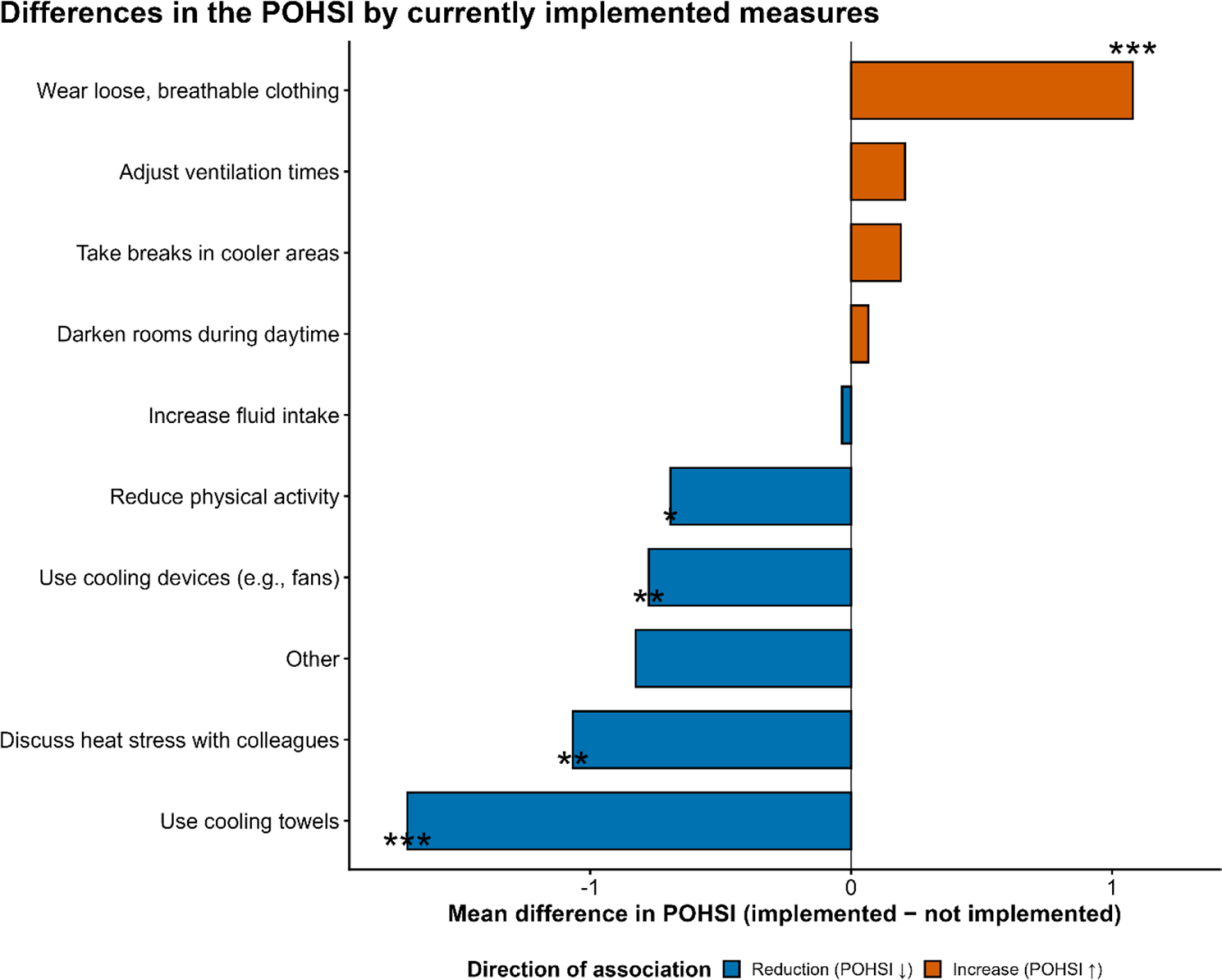
Differences in the Perceived Occupational Heat Stress Index (POHSI) between participants who did and did not implement heat-mitigation measures. Bars represent the mean difference in POHSI (implemented − not implemented); negative values indicate lower perceived heat stress among participants implementing the measure. Asterisks denote statistical significance (*p < 0.05, **p < 0.01, ***p < 0.001)

Lower POHSIs were observed among participants who reported using cooling towels (d ≈ − 0.67), talking to colleagues (d ≈ − 0.37), using cooling devices (d ≈ − 0.27), and reducing physical activity (d ≈ − 0.24), with all p-values < 0.05 (Fig. 3). Higher POHSIs were observed among participants reporting wearing loose or airy clothing (d ≈ 0.36, *p* < 0.05). No significant associations were found for other measures.

Desired preventive measures were predominantly technical and structural measures. Air conditioning (74.4%), ventilation systems (62.4%), and drinking water dispensers (57.0%) were selected most frequently. Organizational measures such as cool breaks (35.7%) and adjusted working hours (25.7%) were also reported. No statistically significant differences in desired measures were observed by gender or occupational category.

### Training and knowledge

Overall, most participating employees reported feeling moderately informed (31%) about health risks related to heat and appropriate coping strategies, while approximately one third indicated that they felt poorly (18%) or not at all informed (15%). Self-reported levels of knowledge did not differ significantly by age group, sex or occupational category.

**Fig. 4:**
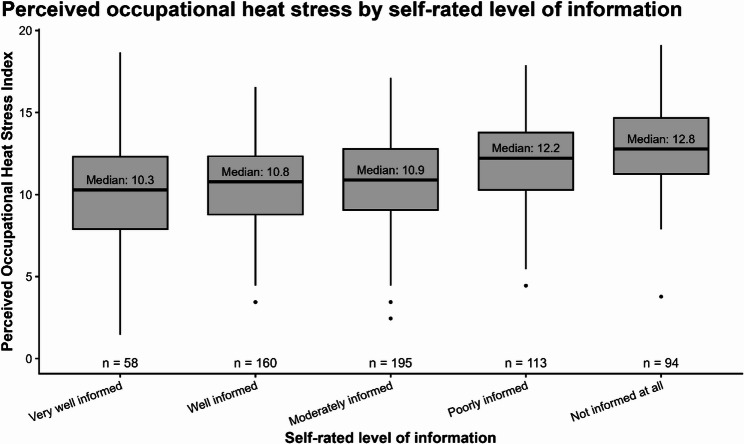
Perceived occupational heat stress by self-rated level of information on heat-related risks and prevention. Boxplots display the distribution of the Perceived Occupational Heat Stress Index (POHSI) across information levels. Horizontal lines indicate median values, which are also shown numerically above each box. Sample sizes (n) for each group are displayed below the corresponding boxplots

POHSI differed significantly across self-reported knowledge and training level (Fig. 4). Participants who felt less informed exhibited higher POHSIs compared with those who reported being well or very well informed. This association was statistically significant in both parametric and non-parametric analyses (ANOVA: F = 15.33, *p* < 0.001; Kruskal–Wallis: χ² = 54.05, *p* < 0.001) with moderate effect sizes (η² ≈ 0.09). Post hoc comparisons confirmed significantly higher POHSIs among participants reporting being poorly or not at all informed compared with better-informed groups.

The number of heat-related preventive measures implemented by participants differed slightly across knowledge categories (ANOVA: F(4, 594) = 2.58, *p* = 0.036), with small effect sizes.

Responses to the open-ended question on information sources showed considerable heterogeneity. Most frequently, participants reported receiving no information. Among those who received information, internet based self-directed research, social and general media predominated. Formal education or training, internal hospital communication, and official sources were mentioned less often. Some respondents reported relying primarily on colleagues or personal experience.

### Heat warning systems

**Fig. 5:**
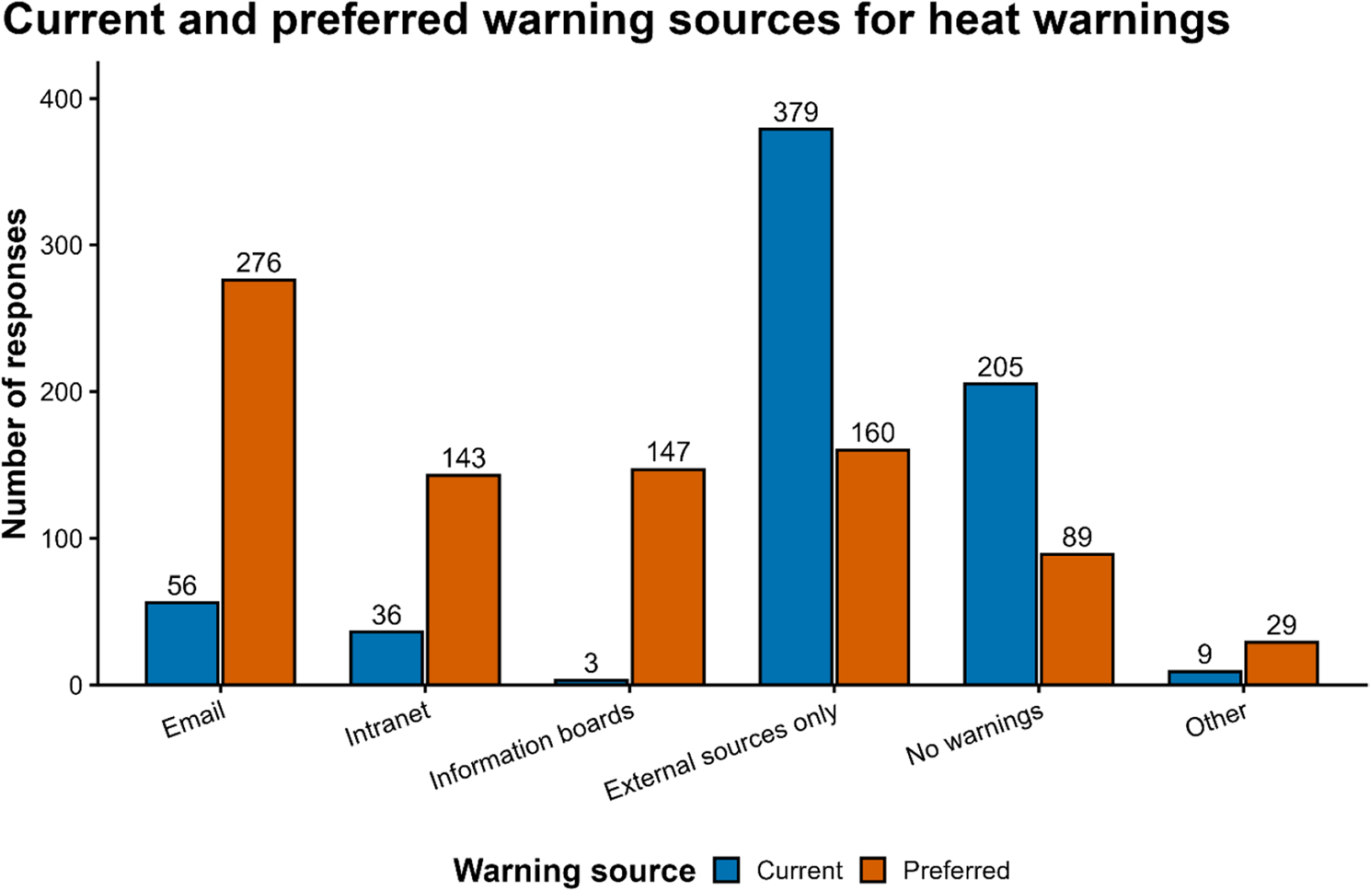
Current and preferred sources of warnings for heat-related risks among participants. Bars show the number of respondents reporting each warning source as currently used or preferred

Most respondents reported receiving heat warnings primarily from external sources, whereas warnings disseminated via internal hospital channels were rare (Fig. 5). A substantial proportion of participants reported not receiving any heat warnings. Reported warning sources did not differ significantly by age, sex or occupational group.

POHSI varied significantly by warning source. Participants reporting the use of external warning sources showed higher POHSIs than those who did not (t-test, *p* < 0.001, Cohen’s d = 0.299). In contrast, participants reporting no heat warnings exhibited significantly lower POHSIs (*p* < 0.001, Cohen’s d = − 0.348). The number of implemented preventive measures differed modestly by warning source (S5 in Supplements). Participants reporting intranet-based warnings implemented more preventive measures than those who did not (*t*(37.8) = 4.77, *p* < 0.001, *d* = 0.78), whereas differences were small for other warning sources.

When asked about preferred channels for receiving heat warnings, participants most frequently selected work-related email (*n* = 277), followed by external sources (*n* = 161), information boards (*n* = 147) and the hospital intranet (*n* = 143), see Fig. 5. Preferences did not differ significantly by age or sex. However, non-clinical participants more frequently preferred receiving heat warnings via work-related email compared with clinical staff (54% vs. 39%; χ² = 12.5, *p* < 0.001, Cramér’s V = 0.136). Clinical staff selected information boards more frequently (26.4% vs. 18.3%; χ² = 5.18, *p* = 0.027, Cramér’s V = 0.08).

### Temperatures and hotspots in the hospital building

Across all sensor boxes mean indoor air temperature, calculated over the entire measurement period, ranged from 22.0 to 25.7 °C (standard deviations 0.8–2.7 °C). Observed extremes reached 10.2 to 33.6 °C between 15th May and 15th October in 2024 and 2025, see S6 in the Supplements. Mean relative humidity varied more strongly among boxes ranging from 40.5 to 76.5% (standard deviations 6.6–24.2%). Since the indoor humidity levels were in a range comfortable for human health and did not add to the temperature-related heat exposure, further analyses focused on temperature alone to define heat exposure hotspots.

The survey indicated that on the 8th, 10th and 12th floor of the Central Building, 50–65% of participants had high or very high POHSIs. In the MCC 56% of respondents had POHSIs that ranged high or very high, and in the Service Building at ground floor level about 77% of respondents fit into this POHSI category. Moderate POHSIs were predominant on the lower floors of the Central Building (40-60%) and the MCC (67%). Low or negligible POHSIs were found in basements of various buildings and smaller administrative or technical buildings.

Based on the distribution of box-wise mean indoor temperatures, sensor boxes were stratified into relative temperature quartiles to examine spatial variation (see S6 in Supplements). The lower quartile included five boxes located primarily on the ground floor and in the north- and west-facing areas of the 12th floor, with mean temperatures ranging from 22.0 to 23.5 °C. The middle quartiles included seven boxes, exhibiting mean temperatures between 23.5 and 25.0 °C. The upper quartile included five boxes, predominantly south-facing on the 11th and 12th floors and the ground floor west of MCC, with mean temperatures ranging 25.1–25.7 °C.

**Fig. 6:**
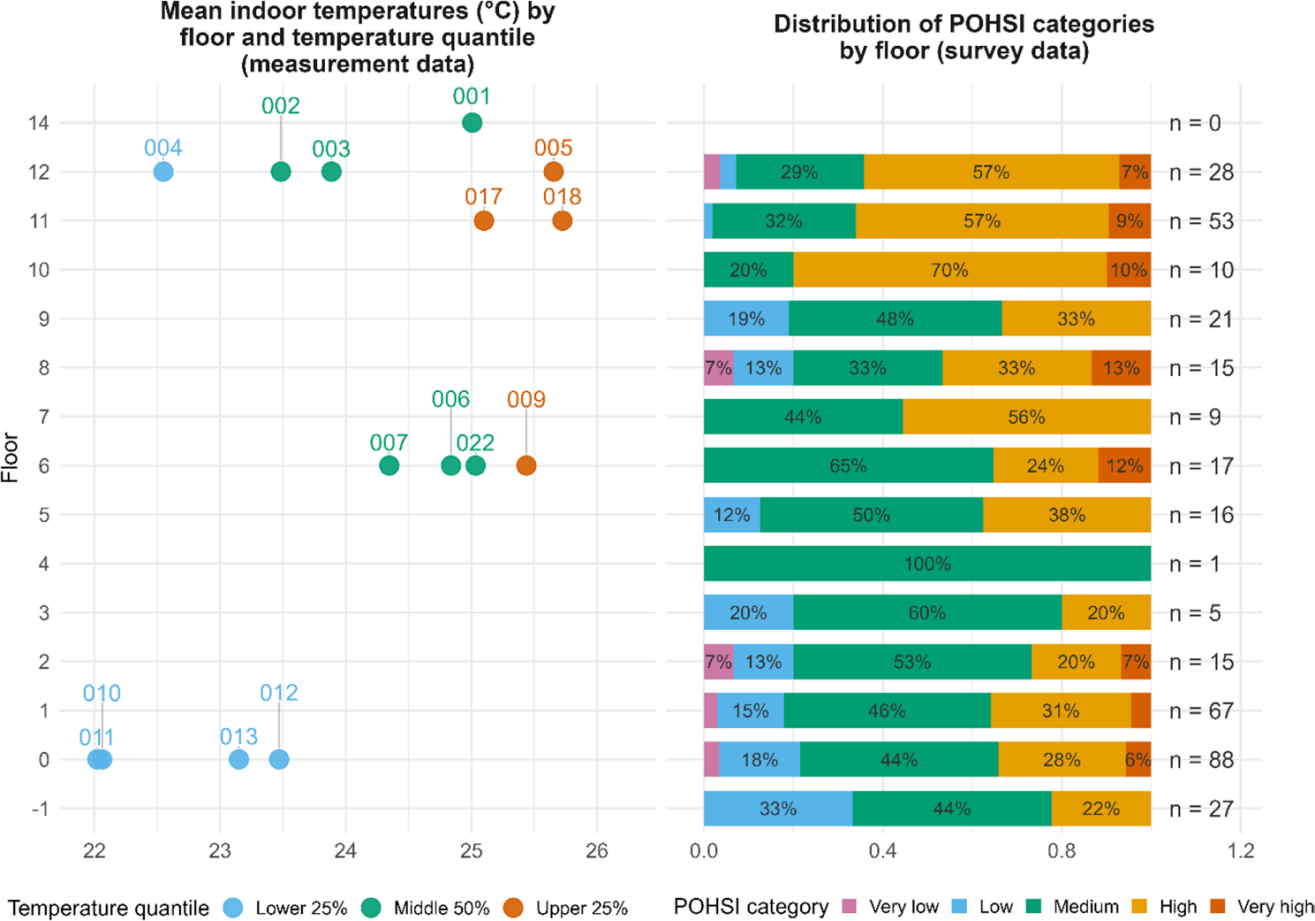
Mean indoor temperatures and perceived occupational heat score index (POHSI) by floor in the main hospital building: The left panel shows the mean indoor temperature measurements by floor and temperature quantile. Numbers indicate the identification codes of the corresponding boxes. The right panel shows the distribution of POHSI categories by floor based on a staff survey. Bars represent proportions of responses per category (stacked to 100%); percentages below 5% are not shown. Absolute numbers of survey responses per floor (n) are indicated

Spatial patterns of survey-based POHSIs corresponded closely with sensor-derived temperature quartiles, as shown in Fig. 6.

## Discussion

### Heat stress, symptoms, and work performance in hospitals

The findings demonstrate that occupational heat stress in hospitals is associated with a broad spectrum of heat-related symptoms and substantial work impairment. The reported symptoms reflect established physiological and cognitive responses to heat exposure [2, 8, 10]. These symptoms are known to impair alertness, decision-making and physical performance [8, 9, 26, 27], all of which are critical for patient safety and quality of care in hospital environments, reinforcing the relevance of occupational heat stress in hospital settings [20].

The pronounced work impairment among participating clinical and physically active staff highlights that heat exposure excessively affects roles critical to patient care. This aligns with previous studies showing that healthcare workers engaged in direct patient care experience higher heat stress due to physical activity, time pressure and personal protective equipment [20, 28].

Together, these findings position heat exposure as an occupational health risk, with implications extending beyond individual discomfort to worker health, productivity patient safety and economics [5, 12, 20]. Ongoing and projected increase in frequency, duration and intensity of heat waves due to climate change, highlight the importance of targeted heat prevention and adaptation measures in hospitals [29, 30].

### Role of knowledge, training, and warning systems

Knowledge and training were identified as important determinants of perceived occupational heat stress among participating hospital employees. Lower levels of knowledge and training were associated with higher perceived occupational heat stress, suggesting that informational gaps increase occupational heat burden. This finding is consistent with health prevention research showing that awareness, risk perception, and knowledge influence both symptom reporting and the ability to adopt effective protective behaviors [19, 24].

Heat warnings were predominantly accessed through external sources such as the media or internet platforms, with minimal reliance on hospital-based internal systems. Reliance on external information contrasts with recommended heat action plans, which emphasize timely, workplace-specific communication integrated into organizational routines [9, 29, 30].

Rather than indicating a protective effect, the association between warning sources and POHSI appears to reflect differences in awareness and risk perception. Participants reporting higher POHSI were more likely to seek information from external warning sources, consistent with evidence that individuals who perceive greater heat-related risk engage more actively in information-seeking behavior [16, 31]. The absence of significant group differences by age, sex, or occupation suggests that current warning practices may not effectively reach staff groups with varying exposure profiles.

A noticeable discrepancy was observed between actual and preferred warning channels. Participants expressed a strong preference for work-related communication channels, particularly email, intranet and information boards. This stresses an underutilized opportunity for hospitals to implement low-threshold, institution-specific heat warning systems that align with staff preferences and support prevention. Integrating heat warnings into existing communication structures represent a feasible and effective strategy to improve heat preparedness in hospitals [32].

### Effectiveness of preventive measures and structural determinants of heat stress

This study provides insight into the perceived effectiveness of heat-related preventive measures implemented by participating hospital employees. Several individual measures were associated with lower POHSIs, including the use of cooling towels, reducing physical activity, and discussing heat-related challenges with colleagues. These findings align with previous research indicating that workload adjustments can reduce perceived heat stress [32, 33].

Not all commonly used measures were associated with reduced heat stress, highlighting important limitations of individual-level prevention under conditions of sustained indoor heat exposure. Wearing loose or airy clothing, for example, was linked to higher POHSI. While the relationship between heat perception and hospital clothing remains underexplored, evidence on fabric-related heat mitigation suggests that hospital scrubs could be reconsidered within existing hygiene and safety regulations [34, 35] Similarly, several other frequently reported measures showed no measurable association with POHSI, including adjusting ventilation times, darkening rooms during daytime hours and increasing fluid intake. These findings indicate a limited protective capacity under sustained heat exposure.

Both the limited effectiveness of some individual measures and the counterintuitive associations observed suggest that individual coping strategies alone may be insufficient when heat exposure remains high. Accordingly, clinical and non-clinical participants expressed demand for technical and organizational interventions, particularly air conditioning, improved ventilation and access to cool rest areas. This pattern highlights the constrained ability of hospital participants to mitigate heat stress through individual behavior alone. Similar to Freire et al. [12], the results indicate that individual preventive actions cannot compensate for inadequate infrastructure or organizational constraints, accentuating the need for structural and organizational heat protection in hospitals in line with established thermal comfort standards.

This interpretation is further supported by the alignment of subjective heat perceptions with objective indoor temperature data. The present findings indicate that self-reported heat burden reflects occupational heat stress shaped by environmental conditions, workload intensity, and limited adaptive capacity [4]. Together, these findings reinforce that effective heat prevention in hospitals must primarily address organizational and building-related determinants, while considering indoor heat sources from technical equipment [9, 17].

### Strengths, limitations, and implications

A key strength of this study is the combined assessment of employee-reported heat perceptions with objective indoor climate measurements in a large tertiary care hospital. Beyond its practical findings, this study contributes methodological insights by demonstrating consistency between subjective heat perceptions and objectively measured indoor temperatures in a real-world hospital setting. Prior research, such as by Quartucci et al. [36], has primarily assessed thermal perception and well-being under controlled laboratory conditions, reporting higher subjective heat perception with rising indoor temperatures. By contrast, the present findings extend this evidence to everyday healthcare environments. Sensor-based temperature data provided essential context on relative spatial heat patterns, while subjective data captured the lived experience of exposure. This complementary assessment provides actionable insights into occupational heat stress under routine working conditions and reflects challenges faced by many healthcare facilities with aging infrastructure and limited cooling capacity [14, 32]. The use of a composite POHSI as an internally consistent indicator allowed a differentiated analysis to support targeted prevention strategies. Despite its good internal consistency, the POHSI requires further validation in independent populations.

Nevertheless, the cross-sectional design limits causal explanations. Voluntary participation and low response rate may have introduced selection bias, as employees more affected by heat stress may have been more likely to participate, thereby limiting the representativeness of the findings. The predominance of female participants broadly reflects global and national healthcare workforce compositions, particularly within nursing professions [37, 38] and the overall ratio within the UKA (77.8% female and 22.2% male employees) [39]. Heat-related symptoms, behaviors, and knowledge were self-reported and therefore subject to reporting bias [40]. Environmental measurements were restricted to selected locations and may not capture all microclimatic conditions within the hospital. However, the consistency between subjective heat perceptions and objective indoor temperature patterns supports the internal plausibility of the observed findings.

Despite these limitations, the study provides a robust evidence base to form targeted heat prevention strategies in hospital settings. The findings support the integration of temperature monitoring and staff feedback into institutional heat preparedness efforts and provide a foundation for implementing and evaluating tailored heat action plans in hospitals.

## Conclusion

Occupational heat stress is an increasingly relevant public health concern in hospital settings. This study demonstrates that healthcare workers experience substantial heat-related symptoms and impaired work performance, particularly in areas with elevated indoor temperatures. While individual coping strategies are commonly applied, their effectiveness remains limited under structurally and organizationally constrained working conditions. The findings underscore the need to prioritize structural and organizational heat protection measures, including building adaptations, optimized ventilation and cooling strategies, and institution-specific heat warning systems. In the present hospital setting, the results provide a data-driven basis to guide the planning and implementation of heat mitigation measures and the development of a hospital-specific heat action plan. Future research should focus on the implementation and systematic evaluation of heat action plans in hospitals, including the effectiveness of technical, organizational and educational interventions, to support evidence-based heat adaptation strategies under increasing climate-related heat exposure.

## Supplementary Information

Supplementary Material 1.

## Data Availability

The datasets used and analyzed during the current study are available from the corresponding author on reasonable request.
