# Supplementary material for: Occupational heat stress in hospital settings: a combined assessment of staff perceptions and indoor climate to support heat action planning
Source: BMC Public Health. 2026 Jul 8;26:2077. doi: 10.1186/s12889-026-28452-4 (PMC13344022; doi:10.1186/s12889-026-28452-4)
Supplement: Supplementary file 1 — Supplementary Material 1. [file 12889_2026_28452_MOESM1_ESM.docx]

**Supplementary material**

**S1:** English translation of the questionnaire used to assess perceived heat exposure, health effects and heat mitigation strategies in the hospital work environment.

| **Domain** | **Question** | **Answer type** | **Answer  Options** |
| --- | --- | --- | --- |
| Demographic and work-related characteristics | How old are you? | Single choice | 16–25 years; 26–35 years; 36–45 years; 46–55 years; 56–65 years; Over 65 years; Prefer not to say |
|  | What is your gender? | Single choice | Female; Male; Diverse; Prefer not to answer |
|  | Do you work in a clinical role or in a non-clinical role? | Single choice | Clinical role; Non-clinical role |
|  | You work in a clinical role. Which professional group do you belong to? | Single choice | Doctor; Nursing staff; Midwife; Psychotherapist; Physical/Occupational/Movement Therapist; Medical Technical Assistant; Other medical assistant; Research staff; Other |
|  | You work in a non-clinical role. Which professional group do you belong to? | Single choice | Administration; Management/Direction; Logistics; Facility Management; Skilled trades; Information technician; Social worker; Kitchen and service staff; Cleaning staff; Supply and waste management; Teaching staff; Pharmacist; Pharmaceutical Technical Assistant; Physician; Laboratory technician; Laboratory assistant; Research staff; Other |
|  | In which building do you spend most of your working time? | Single choice | Main building; Mother-Child-Center ; Center for High-Performance Radiation Therapy; Children's Cancer Center; Academy; West Extension; Medical Faculty; Technical building; Supply building; Administrative Building 1; Administrative Building 2; Administrative Building 3; Administrative Building 4; Various; Other |
|  | On which floor do you work most of the time? | Single choice | Basement; Ground floor; 1st floor; 2nd floor; 3rd floor; 4th floor; 5th floor; 6th floor; 7th floor; 8th floor; 9th floor; 10th floor; 11th floor; 12th floor; 13th floor; 14th floor; Various; Other |
|  | Please rate your physical activity during work. | Likert-Scale | Sitting only; Mostly sitting; Moderately active; Frequently moving; Very high activity |
| Heat perception and thermal discomfort | Overall, how do you perceive the temperature in your work area during summer? | Likert-Scale | Too hot; Hot; Comfortable; Cold; Too cold; Varies (hot in some areas, comfortable or cold in others) |
|  | Are there specific areas in the hospital where heat tends to be a greater problem? | Single choice | Yes; No |
|  | Are there ventilation options available in your work area? | Single choice | Yes, via windows; Yes, via air conditioning; Yes, via ventilation system; Yes, via fans; No, there is no ventilation; Other |
|  | How often does it feel too hot for you during work in the hospital in summer? | Likert-Scale | Never; Rarely; Occasionally; Often; Always |
|  | How would you rate the impact of heat on your work productivity? | Likert-Scale | Very low impact; Low impact; Moderate impact; High impact; Very high impact |
| Heat-related symptoms and work impairment | Which of the following heat-related health problems have you personally experienced during work in the hospital in summer? | Multiple choice | Dizziness; Headaches; Nausea; Vomiting; Fatigue; Exhaustion; Difficulty concentrating; Confusion; Heart palpitations; Muscle cramps; Excessive sweating; No health problems; Other |
|  | Have you ever been unable to work or had to call in sick due to heat? | Likert-Scale | Never; Rarely (has occurred in recent years); Sometimes (1–2 times per summer); Often (several times per summer); Very often (regular sick leave due to heat); Prefer not to say |
| Heat warning systems and information sources | How are you currently warned when a heat wave is approaching? | Multiple choice | Via work email; On the intranet; On information boards in the hospital; Only through external sources (e.g., national weather service, radio, television, internet); Not at all; Other |
|  | How would you like to be warned when a heat wave is approaching? | Multiple choice | Via work email; On the intranet; On information boards in the hospital; Only through external sources (e.g., national weather service, radio, television, internet); Not at all; Other |
| Knowledge, training and preparedness for heat events | Do you feel sufficiently informed to recognize and manage heat-related health problems in yourself and others? | Likert-Scale | Very well informed; Well informed; Moderately informed; Poorly informed; Not informed at all |
|  | How were you informed to recognize and manage heat-related health problems in yourself and others? | Open-ended response |  |
| Currently implemented and desired preventive measures | How do you personally deal with heat-related discomfort during work in the hospital? | Multiple choice | Taking breaks in cooler areas; Drinking more fluids; Using cooling devices (fans, cooling vests); Wearing loose, breathable clothing; Reducing physical activity; Using cooling cloths or towels; Discussing heat mitigation measures with colleagues; Darken rooms during daytime (e.g., by closing blinds); Adjusting ventilation times (at night and in the morning); Other |
|  | Which measures are you aware of to reduce heat in the workplace? | Multiple choice | Adequate ventilation systems; Air conditioning in all work areas; Regular breaks in cooler areas; Provision of cooling devices (fans, cooling vests); Provision of drinking water dispensers; Adjustment of break times within agreements; Adjustment of working hours within agreements; Adjustment of meal plans; Darken rooms during daytime (e.g., by closing blinds); None of the above; Other |
|  | Have measures already been implemented to reduce heat in the workplace during summer? | Single choice | Yes; No; No response |
|  | Which additional measures to reduce heat in the hospital do you consider useful? | Multiple choice | Adequate ventilation systems; Air conditioning in all work areas; Regular breaks in cooler areas; Provision of cooling devices (fans, cooling vests); Provision of drinking water dispensers; Adjustment of break times within collective bargaining agreements; Adjustment of working hours within collective bargaining agreements; Adjustment of meal plans; Darken rooms during daytime (e.g., by closing blinds); None of the above; Other |
|  | Do you have any additional suggestions to improve the management of heat in the workplace? | Open-ended response |  |

**S2:** Identification codes, floor level, orientation and room use of temperature and humidity data loggers deployed in the study buildings.

| **ID** | **Floor** | **Building** | **Direction** | **Use** |
| --- | --- | --- | --- | --- |
| 001 | 12 | Main building | South | Meeting |
| 002 | 12 | Main building | North | Meeting |
| 003 | 12 | Main building | East | Meeting |
| 004 | 12 | Main building | West | Doctor’s Room |
| 005 | 12 | Main building | South | Nurse’s Room |
| 006 | 6 | Main building | South | Nurse’s Room |
| 007 | 6 | Main building | North | Doctor’s Room |
| 022 | 6 | Main building | East | Doctor’s Room |
| 009 | 6 | Main building | West | Doctor’s Room |
| 010 | 0 | Main building | West | Office |
| 011 | 0 | Main building | South | Office |
| 012 | 0 | Main building | East | Office |
| 013 | 0 | Main building | North | Office |
| 014 | 0 | Mother-Child-Center | No direction | Nurse’s Room |
| 015 | 0 | Mother-Child-Center | South | Office |
| 017 | 11 | Main building | South | Doctor’s Room |
| 018 | 11 | Main building | South | Nurse’s Room |

**S3:** Full demographic characteristics of the study participants.

| **Characteristic** | **N = 681**^1^ |
| --- | --- |
| Age |  |
| 16–25 Years | 65 (9.6%) |
| 26–35 Years | 224 (33%) |
| 36–45 Years | 149 (22%) |
| 46–55 Years | 131 (19%) |
| 56–65 Years | 100 (15%) |
| Over 65 Years | 3 (0.4%) |
| No response | 8 (1.1%) |
| Gender |  |
| Female | 517 (76%) |
| Male | 148 (22%) |
| Divers | 2 (0.3%) |
| Preferer not to answer | 4 (0.6%) |
| No response | 9 (1.3%) |
| Floor |  |
| Basement | 41 (6.0%) |
| Ground floor | 180 (26%) |
| 1^st^ Floor | 146 (21%) |
| 2^nd^ Floor | 32 (4.7%) |
| 3^rd^ Floor | 55 (8.1%) |
| 4^th^ Floor | 4 (0.6%) |
| 5^th^ Floor | 20 (2.9%) |
| 6^th^ Floor | 20 (2.9%) |
| 7^th^ Floor | 10 (1.5%) |
| 8^th^ Floor | 17 (2.5%) |
| 9^th^ Floor | 21 (3.1%) |
| 10^th^ Floor | 11 (1.6%) |
| 11^th^ Floor | 66 (9.7%) |
| 12^th^ Floor | 30 (4.4%) |
| 13^th^ Floor | 0 (0%) |
| 14^th^ Floor | 0 (0%) |
| Various | 18 (2.6%) |
| No response | 9 (1.3%) |
| Building |  |
| Central building | 410 (60%) |
| Mother-Child-Center | 69 (10%) |
| Children’s cancer center | 12 (1.8%) |
| Academy | 8 (1.2%) |
| West wing extension | 44 (6.5%) |
| Medical Faculty | 2 (0.3%) |
| Technical building | 7 (1.0%) |
| Supply building | 27 (4.0%) |
| Administration 1-4 | 33 (4.8%) |
| Various | 6 (0.9%) |
| Other | 45 (6.6%) |
| No response | 16 (2.4%) |
| Occupational Category |  |
| Clinical | 440 (65%) |
| Non-clinical | 234 (34%) |
| No response | 6 (0.9%) |
| Clinical Occupations |  |
| Doctor | 67 (9.9%) |
| Nursing staff | 261 (38%) |
| Midwife | 5 (0.7%) |
| Psychotherapist | 10 (1.5%) |
| Physical/Occupational/Movement Therapist | 16 (2.4%) |
| Medical Technical Assistant | 28 (4.1%) |
| Other medical assistant | 36 (5.3%) |
| Speech therapist | 5 (0.7%) |
| Researcher | 10 (1.5%) |
| Other | 6 (0.9%) |
| No response | 236 (35%) |
| Non-Clinical Occupations |  |
| Administration | 116 (17%) |
| Management/Direction | 16 (2.4%) |
| Logistics | 4 (0.6%) |
| Facility Management | 9 (1.3%) |
| IT | 14 (2.1%) |
| Social Work | 5 (0.7%) |
| Chef/Service | 4 (0.6%) |
| Cleaning | 3 (0.4%) |
| Teacher | 4 (0.6%) |
| Pharmacist | 12 (1.8%) |
| Pharmaceutical Technical Assistant | 5 (0.7%) |
| Physician | 6 (0.9%) |
| Laboratory Technician/Assistant | 9 (1.3%) |
| Researcher | 15 (2.2%) |
| Other | 10 (1.5%) |
| No response | 445 (65%) |
| Physical Activity |  |
| Only sitting | 30 (4.4%) |
| Mostly sitting | 148 (22%) |
| Moderately active | 176 (26%) |
| Frequently moving | 146 (21%) |
| Very high activity level | 171 (25%) |
| No response | 9 (1.3%) |
| ^1^n (%) per category | |


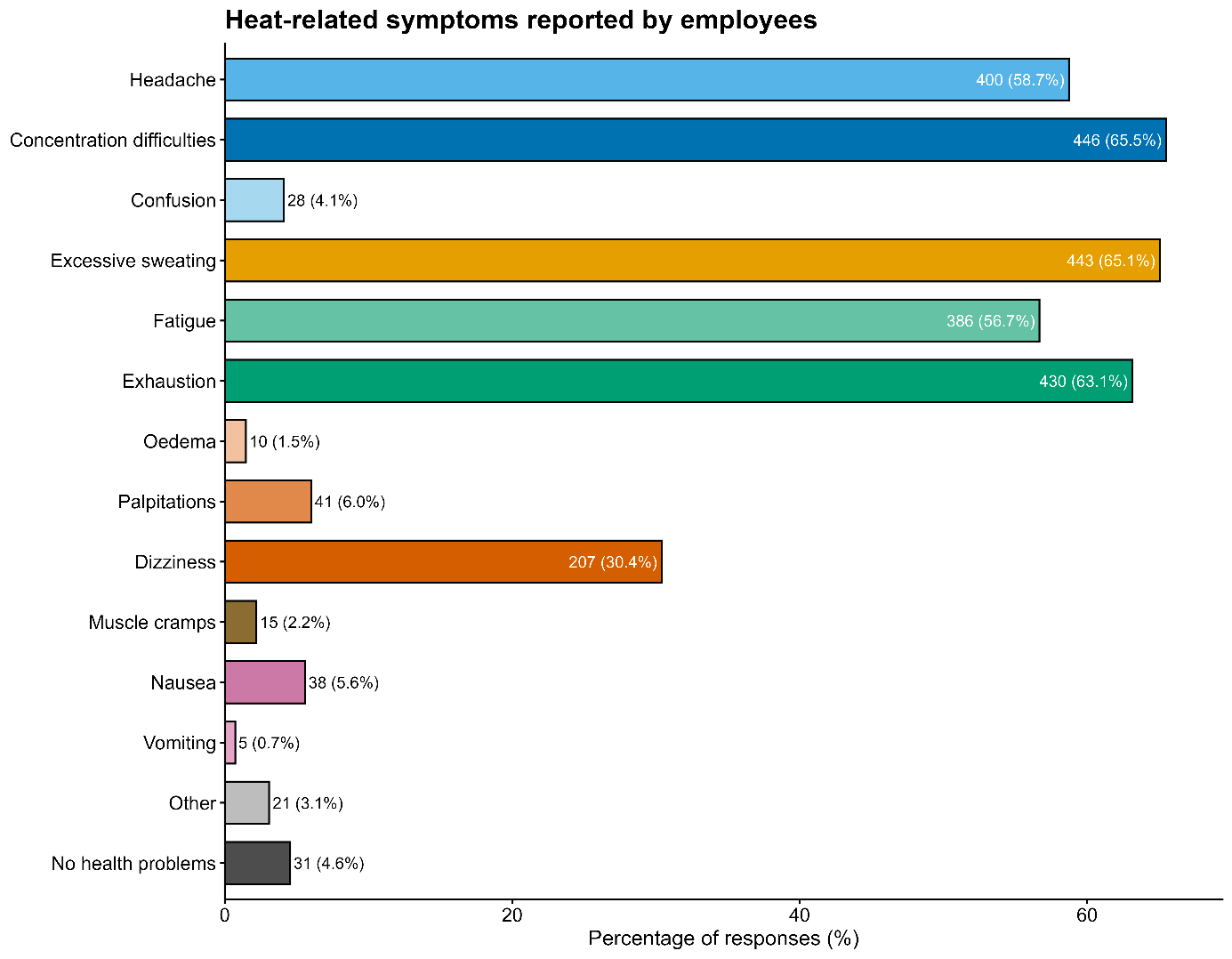


**S4**: Heat-related symptoms reported by employees, grouped into gastrointestinal (vomiting, nausea), musculoskeletal (muscle cramps), cardiovascular (dizziness, palpitations, oedema), fatigue-related (exhaustion, fatigue), thermoregulatory (excessive sweating) and neurological (confusion, concentration difficulties, headache) categories. Bars represent the percentage of responses (n [%]).

**S5:** Number of heat-related preventive measures implemented by warning source

| **Warning source** | **Total (n)** | **Implemented (n)** | **Not implemented (n)** | **t** | **df** | **p** | **Cohen’s d** |
| --- | --- | --- | --- | --- | --- | --- | --- |
| Business email | 607 | 54 | 553 | 2.79 | 62.3 | 0.007 | 0.42 |
| Intranet | 607 | 34 | 573 | 4.77 | 37.8 | <0.001 | 0.78 |
| Information boards | 607 | 3 | 604 | 1.07 | 2.03 | 0.395 | 0.54 |
| External sources | 607 | 369 | 238 | 3.43 | 487 | <0.001 | 0.29 |
| No warnings | 607 | 197 | 410 | -3.84 | 419 | <0.001 | -0.32 |
| Other | 607 | 10 | 597 | 0.01 | 9.2 | 0.989 | 0.01 |

*Differences were tested using independent-samples t tests comparing employees who did versus did not report each warning source. Cohen’s d indicates standardized mean differences; positive values indicate a higher number of implemented preventive measures among employees reporting the warning source, whereas negative values indicate fewer implemented measures*

**S6**: Box-wise mean indoor temperatures and mean relative humidity and their temperature quartile classification with locations. Values are presented as mean ± standard deviation (minimum–maximum). Boxes are stratified into lower 25%, middle 50%, and upper 25% temperature quartiles.

| **ID** | **Location** | **Use** | **Temperature (°C) Mean ± SD  (min–max)** | **Relative humidity (%), Mean ± SD (min–max)** | **Temperature  Quartile Group** |
| --- | --- | --- | --- | --- | --- |
| 011 | Ground floor, south | Office | 25.0 ± 2.0 (18.5–30.7) | 43.1 ± 7.6 (22.3–66.2) | Lower 25% |
| 010 | 1st floor, west | Office | 23.5 ± 2.0 (15.9–28.1) | 76.5 ± 24.2 (35.8–100.0) | Lower 25% |
| 004 | 12th floor, west | Doctor’s Room | 23.9 ± 2.7 (10.2–30.4) | 40.5 ± 7.8 (17.2–80.8) | Lower 25% |
| 013 | Ground floor, north | Office | 22.5 ± 1.8 (12.5–28.4) | 40.8 ± 8.8 (15.0–71.7) | Lower 25% |
| 012 | Ground floor, east | Office | 25.7 ± 1.6 (18.3–30.6) | 41.0 ± 8.5 (14.4–68.8) | Lower 25% |
| 002 | 12th floor, north | Meeting | 24.8 ± 1.1 (21.1–28.7) | 43.3 ± 9.1 (16.5–72.9) | Middle 50% |
| 003 | 12th floor, east | Meeting | 24.3 ± 1.4  (16.8–29.6) | 43.8 ± 8.6 (16.1–72.7) | Middle 50% |
| 015 | Ground floor, no direction (MCC) | Nurse’s Room | 25.4 ± 2.4  (17.4–33.6) | 41.4 ± 8.4 (12.8–63.1) | Middle 50% |
| 007 | 6th floor, north | Doctor’s Room | 22.1 ± 1.6  (15.7–27.4) | 46.3 ± 7.6 (24.0–67.2) | Middle 50% |
| 006 | 6th floor, south | Nurse’s Room | 22.0 ± 1.9  (13.2–27.6) | 48.8 ± 10.8 (21.6–100.0) | Middle 50% |
| 001 | 14th floor, south | Meeting | 23.5 ± 2.0  (11.4–30.8) | 48.5 ± 10.7 (21.2–81.0) | Middle 50% |
| 022 | 6th floor, east | Doctor’s Room | 23.2 ± 1.3  (14.9–28.4) | 56.1 ± 16.9 (26.5–100.0) | Middle 50% |
| 017 | 11th floor, south | Doctor’s Room | 25.4 ± 0.8  (23.7–27.9) | 43.9 ± 9.5 (18.2–64.2) | Upper 25% |
| 014 | Ground floor, west (MCC) | Nurse’s Room | 24.1 ± 1.5  (14.2–31.0) | 48.4 ± 10.8 (17.7–80.3) | Upper 25% |
| 009 | 6th floor, west | Doctor’s Room | 25.1 ± 2.0  (17.6–31.0) | 42.8 ± 7.5 (24.9–74.0) | Upper 25% |
| 005 | 12th floor, south | Nurse’s Room | 25.7 ± 2.1  (18.1–31.8) | 40.8 ± 7.7 (22.7–67.2) | Upper 25% |
| 018 | 11th floor, south | Nurse’s Room | 25.0 ± 1.8  (16.2–30.4) | 40.9 ± 6.6 (24.0–66.5) | Upper 25% |
